# LSD1 promotes prostate cancer cell proliferation by upregulating *PRAC1* expression

**DOI:** 10.1038/s41598-026-42928-8

**Published:** 2026-03-10

**Authors:** Yang Liao, Chuan Liu

**Affiliations:** Department of Urology, The Second Affiliated Hospital of Chongqing Medical University, Chongqing, Chongqing, 400016 P.R. China

**Keywords:** Prostate cancer, Proliferation, PRAC1, LSD1, TAK-418, Cancer, Cell biology, Oncology, Urology

## Abstract

*PRAC1*, which is specifically expressed in prostate, rectal, and distal colon tissues, plays a critical role in the maintenance and self-renewal of prostate epithelial stem cells. However, the role of *PRAC1* in prostate cancer is unclear. In this study, we found that *PRAC1* expression is upregulated in prostate cancer cells and that *PRAC1* knockdown represses the proliferation of prostate cancer cells. Moreover, lysine-specific demethylase 1 (LSD1) promoted prostate cancer cell proliferation by upregulating *PRAC1* expression. TAK-418, an LSD1 inhibitor, suppressed prostate cancer cell proliferation by downregulating *PRAC1* expression. Our results highlight *PRAC1* or LSD1 inhibition as promising avenues for prostate cancer treatment.

**Supplementary Information:**

The online version contains supplementary material available at 10.1038/s41598-026-42928-8.

## Introduction

Prostate cancer is a leading malignancy in men worldwide that contributes substantially to global male mortality. Although its incidence rates vary considerably across regions, with a higher prevalence in developed countries, emerging evidence suggests rising trends in developing nations owing to aging populations and lifestyle changes^1,2^. Prostate cancer poses substantial health risks, particularly for men aged > 50 years, those with a family history of the disease, and individuals of African descent, who face disproportionately higher morbidity and mortality^1,2^. Although prostate cancer often progresses indolently, advanced stages can aggressively metastasize to bones and organs, causing debilitating complications^2,3^. Advancing the understanding of prostate cancer pathogenesis and the identification of genes responsible for its malignant progression hold promise for the development of novel therapeutic targets.

*PRAC1* encodes a 382 nucleotide RNA and a 6-kDa nuclear protein found in the prostate, rectum, and distal colon. The *PRAC1* gene is located on chromosome 17 at position 17q21, approximately 4 kb downstream of the homeodomain of Hoxb-13^4^. Germline mutations in HOXB13 are associated with an increased risk of prostate cancer^5,6^. *PRAC1* mRNA is a specific marker distinguishing semen from other bodily fluids^7^. RNA in situ hybridization with a *PRAC1* mRNA probe enables detection of exfoliated prostate cancer cells in urine^8,9^. Research has shown that *PRAC1* expression is lower in prostate cancer tissues than in benign prostatic hyperplasia tissues^10^ and that *PRAC1* plays an essential role in the maintenance and self-renewal of prostate epithelial stem cells^11^. However, the role of *PRAC1* in prostate cancer remains unclear.

Therefore, we investigated the role of *PRAC1* in prostate cancer in this study. We found that *PRAC1* expression is upregulated in cancerous compared with non-cancerous prostate tissues, and that *PRAC1* knockdown inhibits prostate cancer cell proliferation. Moreover, we found that lysine-specific demethylase 1 (LSD1) promotes *PRAC1* expression in prostate cancer cells and that LSD1 inhibition represses prostate cancer cell proliferation by downregulating *PRAC1* expression. Our findings highlight *PRAC1* as a novel therapeutic target for prostate cancer therapy.

## Results

### ***PRAC1*** knockdown inhibits proliferation, colony formation, migration, and invasion of prostate cancer cells

**Fig. 1:**
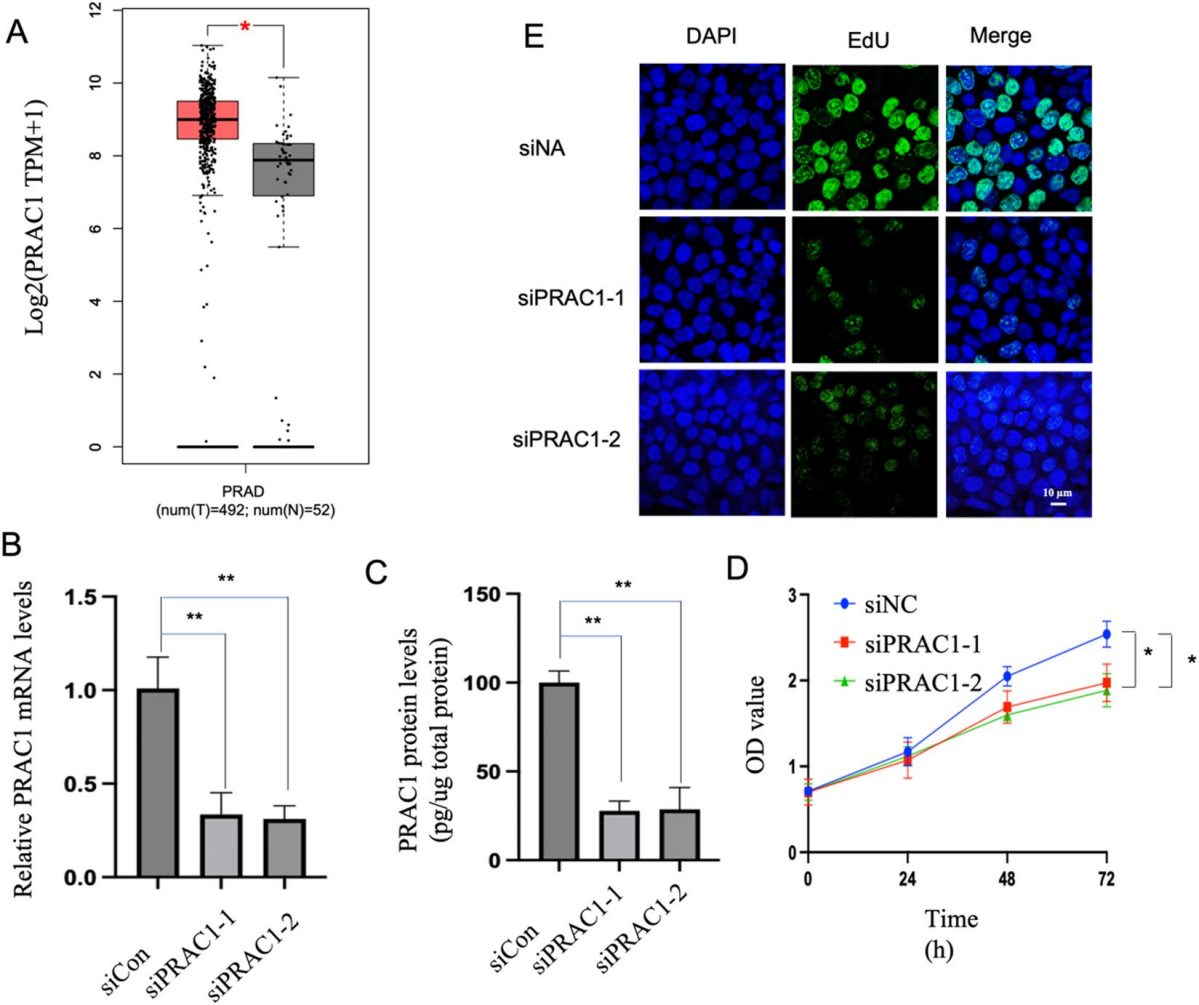
***PRAC1*** **knockdown inhibits prostate cancer cell proliferation.** (A) *PRAC1* mRNA levels in cancerous and non-cancerous prostate tissues. Data were downloaded from The Cancer Genome Atlas. Red: prostate cancer tissue (*n* = 492); gray: non-cancerous prostate tissue (*n* = 52). (B–E) LNCaP cells were transfected with control or *PRAC1* siRNA, and mRNA (B) and protein (C) levels of PRAC1 were determined using reverse transcription quantitative PCR and enzyme-linked immunosorbent assays, respectively, 48 h after transfection. (D, E) Cell proliferation was determined using the CCK8 (D) and EdU incorporation assays (E). Statistical analyses were performed using two-sided Student’s *t*-tests; *p < 0.05, ** *p* < 0.001.

**Fig. 2:**
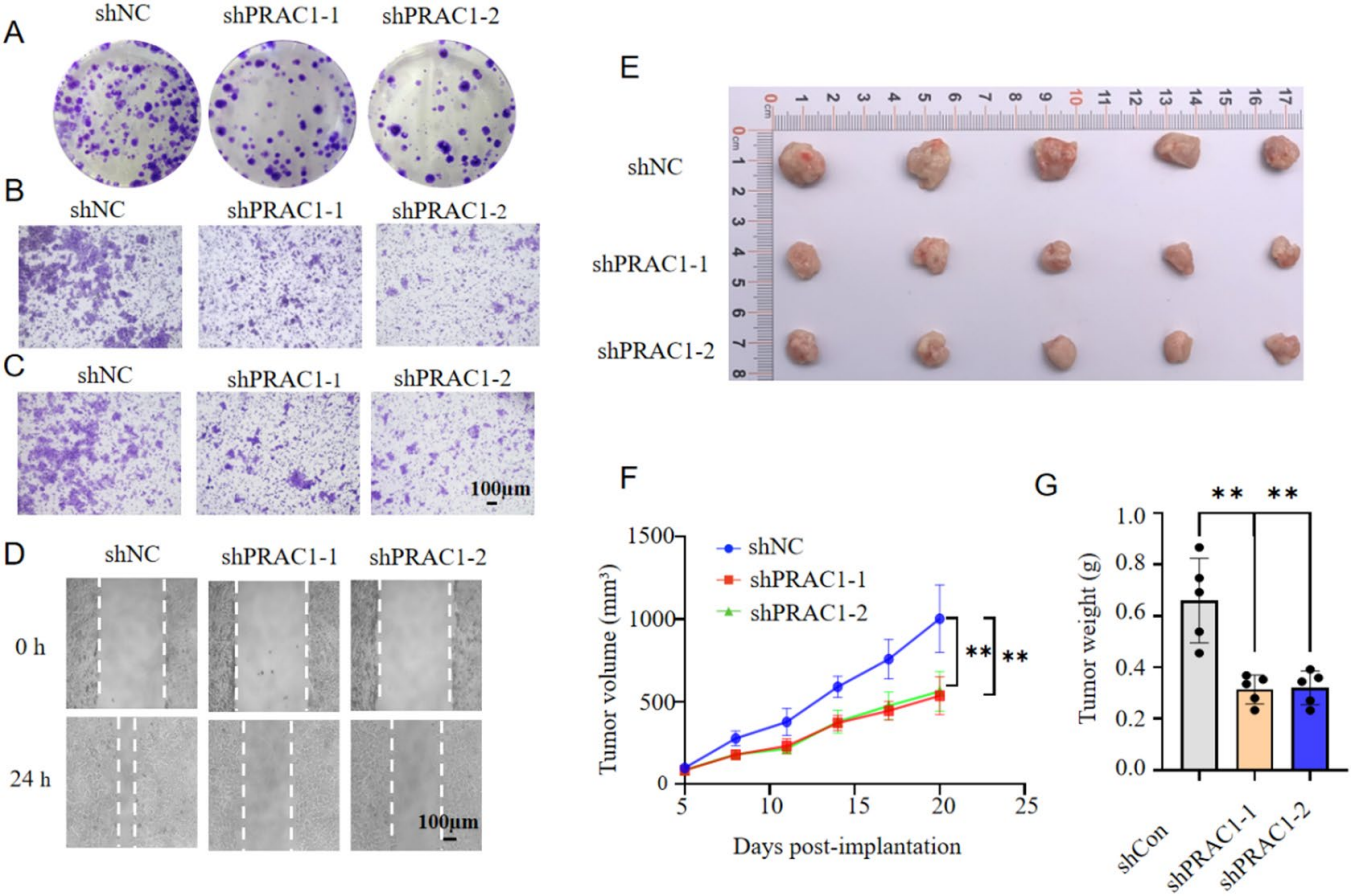
***PRAC1*** **knockdown inhibits colony formation**,** migration**,** and invasion of prostate cancer cells and represses xenograft tumor growth**. (A-C) Colony formation (A), migration (B), and invasion (C) assays of control or *PRAC1* knockdown cells. (D) Wound healing assay of control or *PRAC1* knockdown cells. (E-G) Xenograft tumors of control or *PRAC1* knockdown cells were photographed (E), the average volume (F) and weight (G) were determined. **P < 0.01 (two-sided Student’s t test).

To investigate the role of *PRAC1* in prostate cancer, we compared the expression levels of *PRAC1* in prostate cancer and non-cancerous prostate tissues using The Cancer Genome Atlas and the Genotype-Tissue Expression Portal. *PRAC1* expression was upregulated in cancerous compared with non-cancerous tissues (Fig. 1A). Next, to investigate the effect of *PRAC1* on the proliferation, colony formation, migration, and invasion of prostate cancer cells, we knocked down *PRAC1* in LNCaP and DU145 cells using two specific small interfering RNAs (siRNAs; Fig. 1B–C and Supplementary Fig. 1 A, B). The Cell Counting Kit-8 (CCK-8) assay and 5-ethynyl-2’-deoxyuridine (EdU) incorporation assay revealed that *PRAC1* knockdown reduced cell proliferation (Fig. 1D, E and Supplementary Fig. 1 C). Additionally, we found that *PRAC1* knockdown repressed the colony formation, migration, and invasion of prostate cancer cells (Fig. 2A–D). To further examine the effect of *PRAC1* on prostate tumor growth in vivo, we established LNCaP xenograft mouse models. Our results revealed that *PRAC1* knockdown repressed xenograft tumor growth (Fig. 2E–G).

### **LSD1 promotes*PRAC1*expression in prostate cancer cells**

**Fig. 3:**
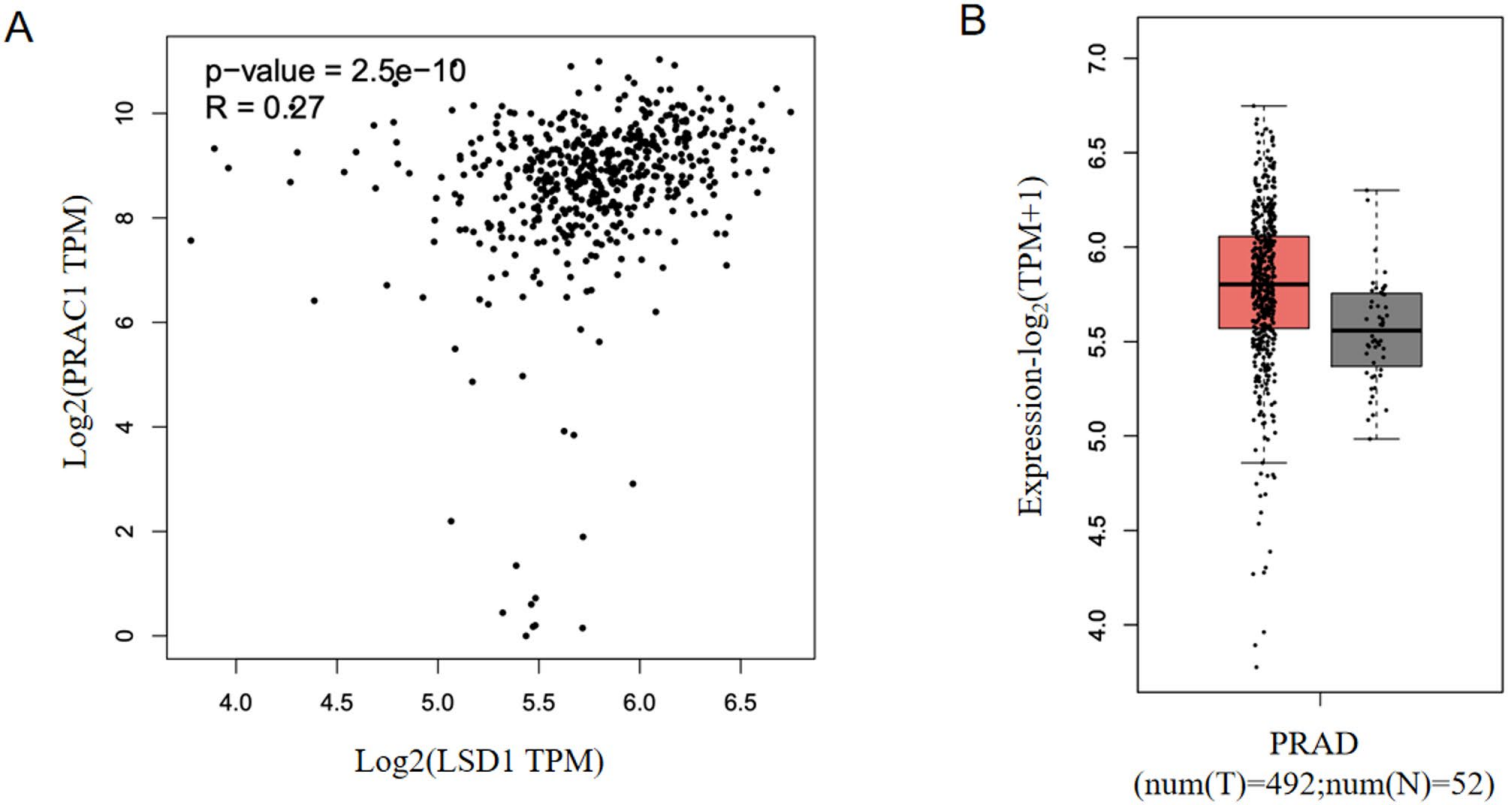
**The expression of** ***LSD1*** **is correlated with that of** ***PRAC1*** **in prostate cancer tissues.** (A) Pearson correlation analysis of *PRAC1* and *LSD1* mRNA expression levels in 492 cancerous and 52 non-cancerous prostate tissues. Data were downloaded from The Cancer Genome Atlas. (B) *LSD1* mRNA levels in prostate cancer tissues and non-cancerous prostate tissues. Data were downloaded from The Cancer Genome Atlas. Red: prostate cancer tissue (*n* = 492); gray: non-cancerous prostate tissue (*n* = 52).

To investigate the mechanism underlying the upregulation of *PRAC1* expression in prostate cancer cells, we searched GPSAdb, a database for exploring transcriptomic consequences of gene perturbations in human cell lines^12^, to identify candidate genes that may regulate *PRAC1* expression. As shown in Table 1, the knockdown of *IQGAP3*, *HSF1*, *HSF2*, *PRRC2B*, *LSD1*, *POU5F1*, *ARPIN*, *RB1*, *ZZZ3*, *TP53*, *EWSR1*, *ALKBH5*, *BUB3*, or *RUVBL1* led to the downregulation of *PRAC1* expression. Additionally, we found that the mRNA expression levels of *PRAC1* were positively correlated with those of LSD1 in cancerous and non-cancerous prostate tissues (Fig. 3A; Table 1) and that the mRNA expression levels of *LSD1* were upregulated in cancerous versus non-cancerous prostate tissues (Fig. 3B). These data suggest that LSD1 may promote *PRAC1* expression in prostate cancer tissues.

**Table 1:** Genes that can regulate *PRAC1* expression.

pbgene	Method	Cell line	logFC	*P* value	*R value
***IQGAP3***	siRNA	NTERA-2 cl.D1	−8.184598395	9.16E-12	0.03
***HSF1***	siRNA	MCF 7.00	−5.067259342	3.41E-12	−0.2
***HSF2***	siRNA	MCF 7.00	−4.474306955	2.60E-05	−0.28
***PRRC2B***	shRNA	HEK293T	−3.979308572	3.44E-05	−0.15
***LSD1***	shRNA	A-673	−3.801029854	2.44E-121	0.27
***POU5F1***	shRNA	iPSCs	−3.787135872	1.15E-05	−0.3
***ARPIN***	shRNA	LoVo	−3.173206952	1.95E-08	0.089
***RB1***	shRNA	PC-3	−3.043327119	1.58E-06	−0.14
***ZZZ3***	siRNA	WA17	−2.609872037	2.89E-11	−0.04
***TP53***	shRNA	LNCaP clone FGC	−2.574998038	3.52E-142	0.083
***RB1***	shRNA	LNCaP C4-2	−2.39566212	1.92E-97	−0.14
***EWSR1***	siRNA	A-673	−2.138551717	4.62E-50	−0.24
***ALKBH5***	ko	hESCs	−2.126864494	5.03E-09	−0.16
***BUB3***	shRNA	LNCaP	−2.118112727	1.97E-30	0.06
***EWSR1***	siRNA	A-673	−2.082121605	6.99E-05	−0.24
***RUVBL1***	shRNA	BPH-1	−2.061111305	1.22E-19	0.089

* Correlation between the expression of *PRAC1* and that of the indicated genes in cancerous and non-cancerous prostate tissues.

**Fig. 4:**
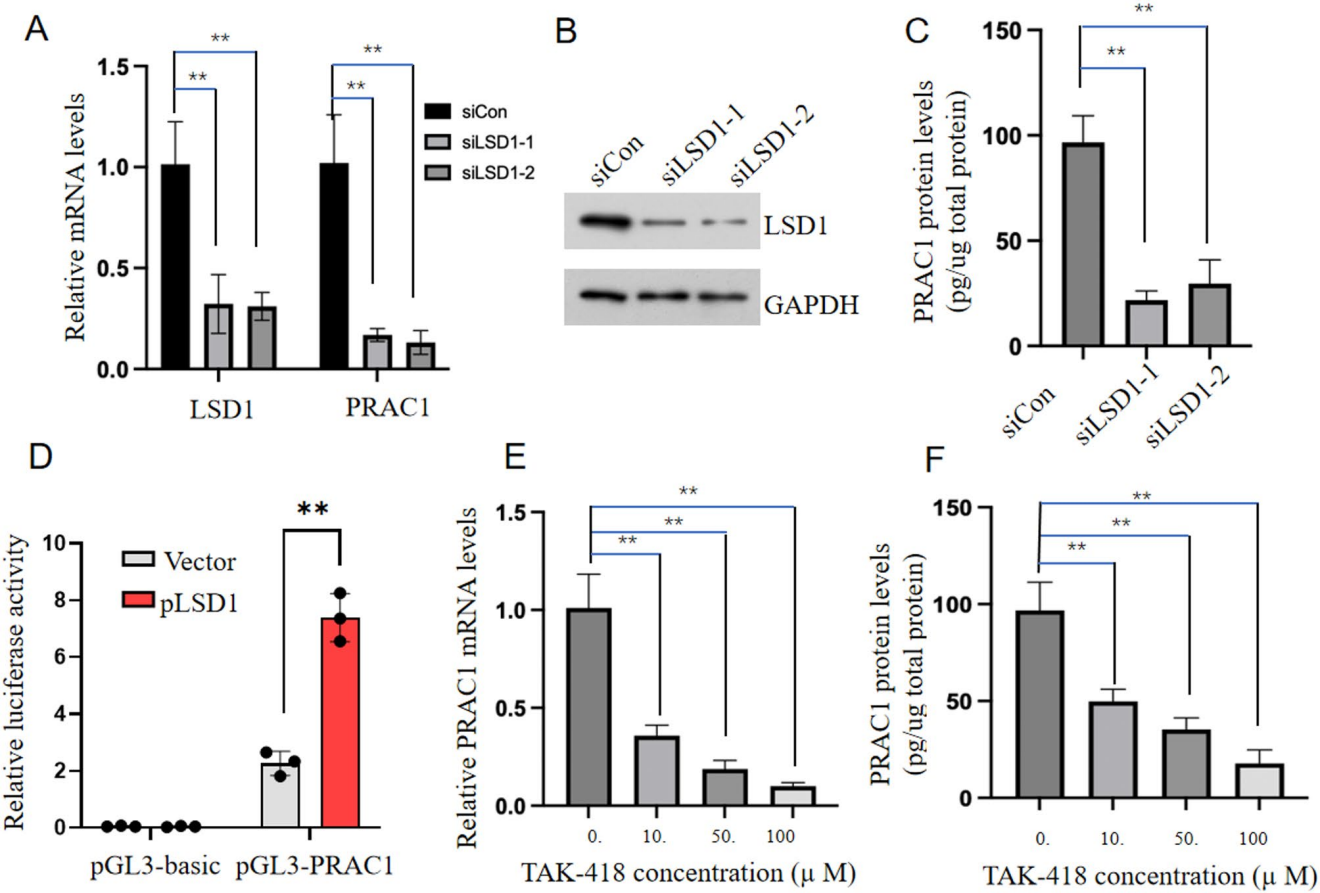
**LSD1 promotes** ***PRAC1*** **expression in prostate cancer cells.** (A–C) LNCaP cells were transfected with control or *LSD1* siRNAs. (A) *LSD1* and *PRAC1* mRNA levels were determined using reverse transcription quantitative PCR 48 h after transfection, (B) LSD1 protein levels were determined using western blotting 48 h after transfection, and (C) PRAC1 protein levels were determined using an enzyme-linked immunosorbent assay 48 h after transfection. (D) Luciferase activities of the PRAC1 promoter reporter in LNCaP cells transfected with a LSD1 expression plasmid or control plasmid. (E, F) PRAC1 mRNA (E) and protein (F) levels in LNCaP cells were determined using reverse transcription quantitative PCR and an enzyme-linked immunosorbent assay, respectively, 72 h after treatment with different doses of TAK-418. **P < 0.01 (two-sided Student’s t test).

To investigate the regulatory role of LSD1 in *PRAC1* expression, we knocked down *LSD1* in prostate cancer cells, which inhibited *PRAC1* expression (Fig. 4A–C and Supplementary Fig. 2A–C). Additionally, we found that LSD1 overexpression enhanced the promoter activity of *PRAC1* and promoted *PRAC1* expression (Fig. 4D). Furthermore, treatment with TAK-418, a specific LSD1 inhibitor, suppressed *PRAC1* expression in a dose-dependent manner (Fig. 4E, F and Supplementary Fig. 2D, E). Taken together, these data indicate that LSD1 promotes *PRAC1* expression in prostate cancer cells.

### **TAK-418 represses prostate cancer cell proliferation by inhibiting*PRAC1*expression**

**Fig. 5:**
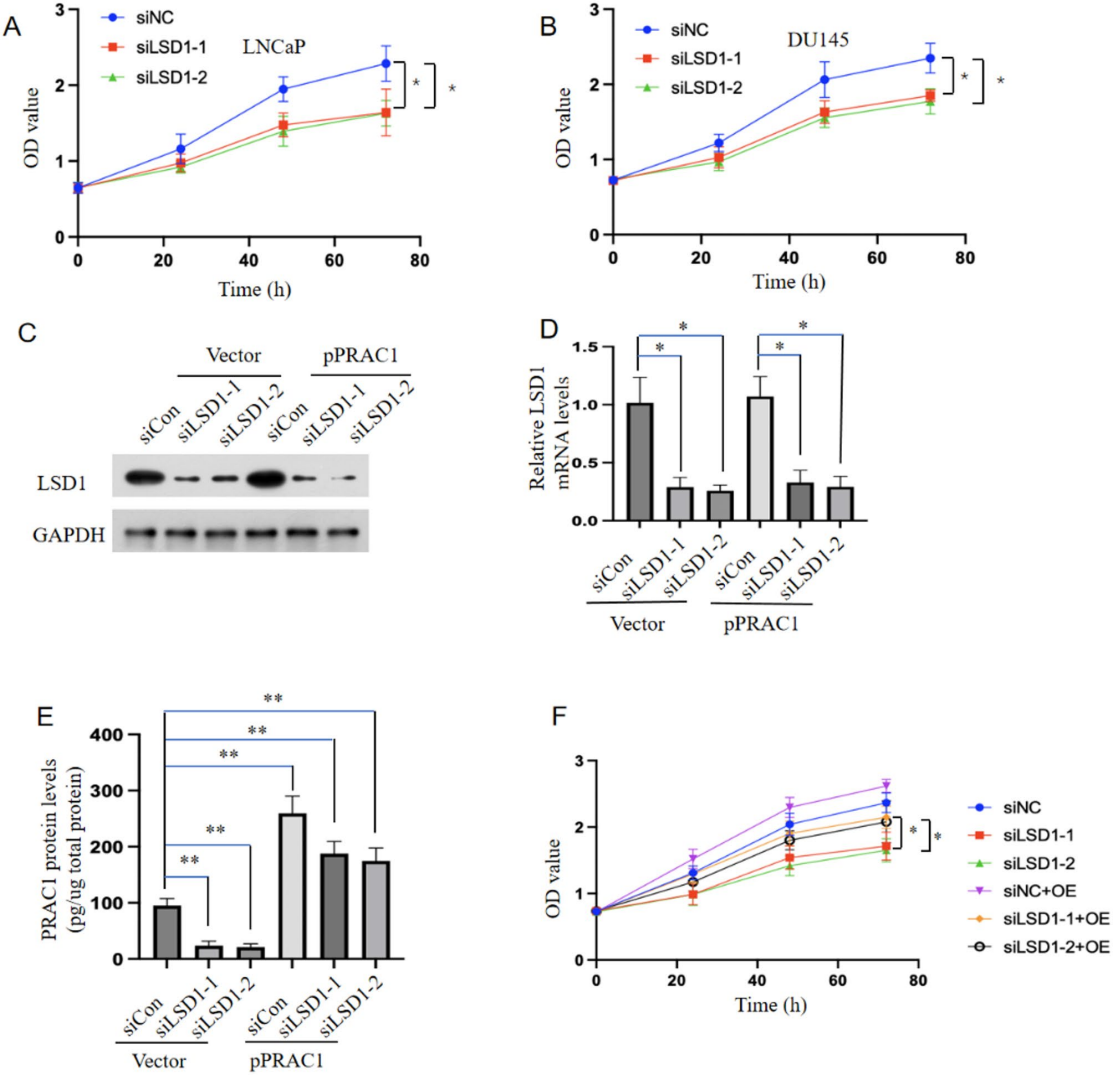
***LSD1*** **knockdown represses prostate cancer cell proliferation by inhibiting** ***PRAC1*** **expression.** LNCaP (A) and DU145 (B) cells were transfected with control or *LSD1* siRNAs, and cell proliferation assays were performed at different time points after transfection. (c–f) LNCaP cells were transfected with *LSD1* siRNAs or co-transfected with *LSD1* siRNAs and *PRAC1*-expressing plasmid; LSD1 protein (C) and mRNA (D) levels were determined using reverse transcription quantitative PCR and western blotting, respectively, at 48 h after transfection. (E) Protein levels of PRAC1 were determined using an enzyme-linked immunosorbent assay 48 h after transfection, and (F) cell proliferation assays were performed at different time points after transfection. Statistical analysis was performed using two-sided Student’s *t*-tests; *p < 0.05, ** *p* < 0.001.

**Fig. 6:**
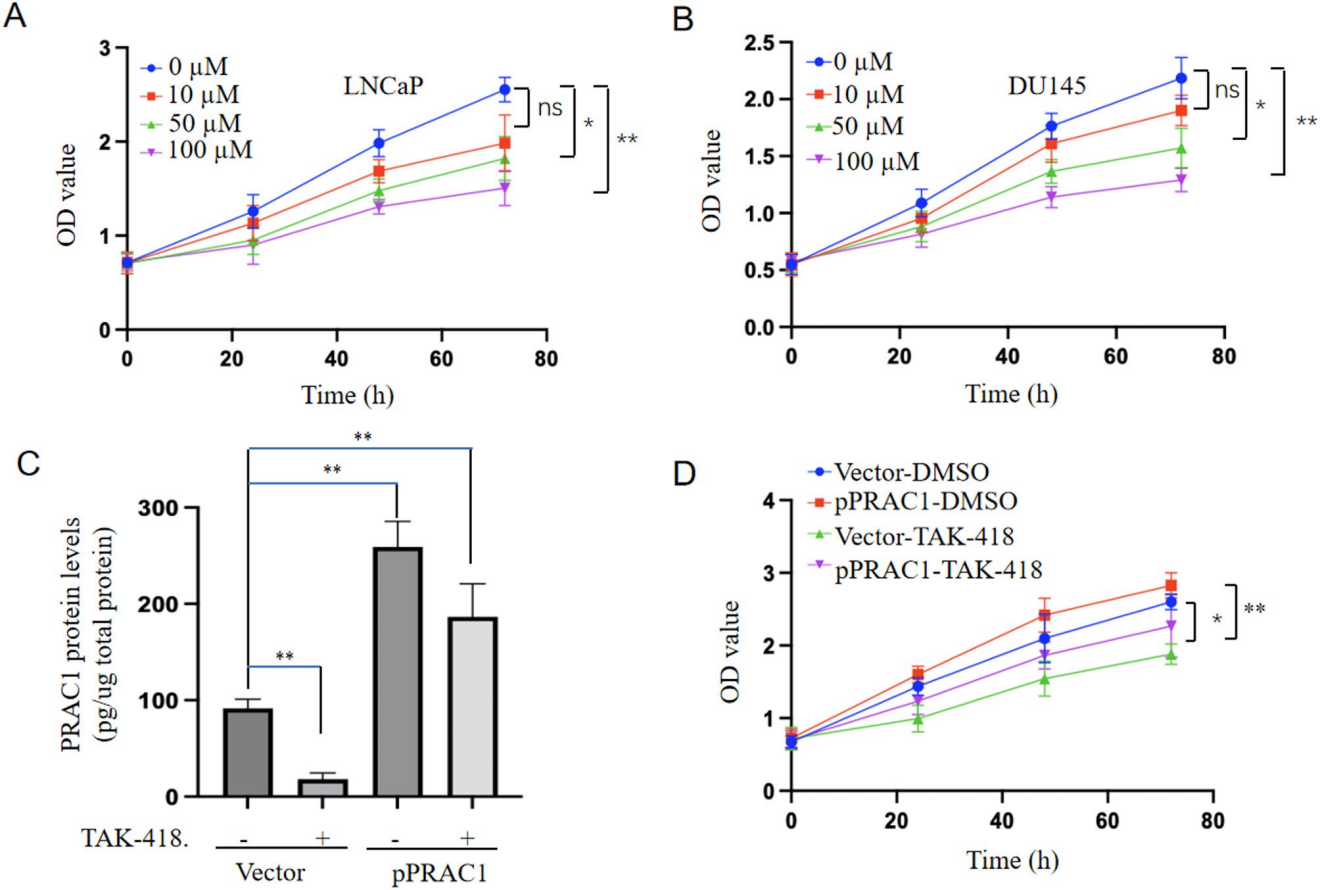
**TAK-418 represses prostate cancer cell proliferation by inhibiting** ***PRAC1*** **expression.** LNCaP (A) and DU145 (B) cells were treated with different concentrations of TAK-418, and cell proliferation assays were performed at different time points. (C, D) LNCaP cells were transfected with *PRAC1*-expressing plasmid or control plasmid and treated with or without 100 µM TAK-418. (C) Protein levels of PRAC1 were determined using enzyme-linked immunosorbent assay. (D) Cell proliferation assays were performed at different time points after transfection. Statistical analysis was performed using two-sided Student’s *t*-tests; *p < 0.05, ** *p* < 0.001.

Given that LSD1 promotes the expression of *PRAC1* and that inhibiting *PRAC1* expression suppresses the proliferation of prostate cancer cells, we investigated whether LSD1 inhibition can repress prostate cancer cell proliferation by suppressing *PRAC1* expression. The specific knockdown of *LSD1* in prostate cancer cells significantly inhibited cellular proliferation (Fig. 5A–D), whereas *PRAC1* overexpression partially rescued this effect (Fig. 5E, F). Moreover, treatment with TAK-418 repressed prostate cancer cell proliferation (Fig. 6A, B); *PRAC1* overexpression partially rescued this effect (Fig. 6C, D).

## Discussion

*PRAC1* is specifically expressed in prostate, rectal, and distal colon tissues and plays a critical role in the maintenance and self-renewal of prostate epithelial stem cells. Notably, *PRAC1* mRNA serves as a semen-specific biomarker for forensic identification, enabling the non-invasive detection of exfoliated prostate cancer cells in urine^4,7–9^. In this study, we found that *PRAC1* expression is upregulated in prostate cancer cells, and that *PRAC1* knockdown represses prostate cancer cell proliferation. These findings identify *PRAC1* as a potential therapeutic target in prostate cancer.

LSD1 is an evolutionarily conserved histone demethylase that dynamically regulates the chromatin structure and gene transcription by removing methyl groups from histone H3 lysine residues at positions 4 (H3K4) and 9 (H3K9) via oxidative reactions. As a core component of multiprotein complexes (e.g., CoREST/histone deacetylase complexes), LSD1 mediates transcriptional repression via H3K4 demethylation and participates in stem cell differentiation, embryonic development, and hematopoietic cell maturation^10,13–16^. In nuclear receptor signaling pathways such as androgen receptor signaling, LSD1 removes repressive H3K9 methylation marks to activate target gene transcription and promote prostate cancer cell proliferation^17,18^. LSD1 also exhibits non-histone substrate specificity, regulating the methylation status of proteins such as p53 and DNMT1 to influence DNA damage response and methylation homeostasis^17,19,20^. The activity of LSD1 is dynamically regulated via post-translational modifications and combinatorial associations with corepressors, such as ZNF198 or REST^21,22^. By integrating epigenetic modifications with signaling networks, LSD1 serves as a key spatiotemporal regulator of gene expression. Aberrant LSD1 activity contributes to tumorigenesis, viral latency, and other pathological processes^11,20,23^. LSD1 also promotes cancer progression via multiple epigenetic and non-epigenetic mechanisms^24^. In this study, we demonstrated that LSD1 promotes prostate cancer cell proliferation by upregulating *PRAC1* expression.

TAK-418 is a reversible brain-penetrating inhibitor that selectively targets LSD1 to block histone H3K4me1/me2 demethylation. Structurally optimized to minimize the off-target effects on monoamine oxidases, TAK-418 exhibits superior safety to earlier LSD1 inhibitors, avoiding hematological toxicity^25–28^. Although primarily investigated in neurodevelopmental disorders^26,27,29–31^, the ability of TAK-418 to modulate cancer-related epigenetic plasticity makes it a promising candidate for precision oncology, particularly in tumors with LSD1 amplification or aberrant chromatin remodeling. In solid tumors, TAK-418 synergizes with DNA-demethylating agents or histone deacetylase inhibitors to enhance epigenetic reprogramming and overcome resistance to therapy^32,33^. Early-phase trials explored the use of TAK-418 in combination with immune checkpoint inhibitors, leveraging LSD1 inhibition to enhance tumor immunogenicity by upregulating endogenous retroviral elements and antigen presentation^32^. We found that TAK-418 inhibits prostate cancer cell proliferation by repressing *PRAC1* expression.

In conclusion, we found that LSD1 promotes prostate cancer cell proliferation by upregulating *PRAC1* expression. Therefore, we propose *PRAC1* or LSD1 inhibition as a promising therapeutic avenue for prostate cancer treatment.

## Materials and methods

### Cell culture and treatment

LNCaP and DU145 cells were obtained from the National Collection of Authenticated Cell Cultures (Shanghai, China) and cultured in Roswell Park Memorial Institute (RPMI) 1640 medium supplemented with 1% antibiotics (100 U/mL penicillin and 100 µg/mL streptomycin sulfate; Sigma-Aldrich, Burlington, MA, USA) and 10% fetal bovine serum (FBS; Gibco, Waltham, MA, USA). The cells were maintained at 37 °C in a humidified incubator with 5% CO2. To inhibit LSD1 activity, the cells were exposed to different concentrations of TAK-418 (MedChemExpress, Monmouth Junction, NJ, USA). To induce apoptosis, the cells were treated with 0.5 µM Docetaxel (MedChemExpress).

## Plasmids and siRNA transfection

The siRNAs were purchased from General Biol (Anhui, China). Cells were transfected with siRNAs using Lipofectamine 2000 (Life Technologies, Carlsbad, CA, USA) according to the manufacturer’s instructions. The siRNA sequences were as follows: si-PRAC1-1: 5’-CATCTTACTACCTCCAAGAGT-3’; si-PRAC1-2:5’-GCTCAGCCTGTAATTCTGGAA-3’; siLSD1-1:5’-TGAATTAGCTGAAACACAATT-3’; siLSD1-2:5’-GCCTAGACATTAAACTGAATA-3’.

Plasmid transfection was performed using Lipofectamine 8000 (Life Technologies, Carlsbad, CA, USA) according to the manufacturer’s instructions. The PRAC1 or LSD1 expression plasmid was engineered by cloning the full-length open reading frame of PRAC1 or LSD1 into the pCDH-CMV-MCS-EF1-Puro lentiviral vector. The shPRAC1-1 and shPRAC1-2 plasmids were generated by cloning hairpin oligonucleotides into the pLVX-shRNA2-puro vector, with targeting sequences identical to those of siPRAC1-1 and siPRAC1-2, respectively.

## Colony formation

LNCaP-shNC, LNCaP-shPRAC1-1, and LNCaP-shPRAC1-2 cells were seeded in 6-well plates and cultured for 12 days. Wells were rinsed three times with PBS, and cell colonies were stained with a 0.5% crystal violet solution for 10 min. After air drying at room temperature, the plates were photographed.

### Mouse xenograft tumor model

Female BALB/c athymic nude mice (6–8 weeks old; Vital River Experimental Animal Center, Beijing, China) were subcutaneously injected with 5 × 10^6^ cells to generate tumor xenografts. All animal procedures were approved by the Animal Care Committee of Chongqing Medical University and were performed in accordance with all relevant guidelines and regulations.

## Reverse transcription and quantitative polymerase chain reaction

Total RNA was isolated using TRIzol reagent (Life Technologies), incubated with RNase-free DNase I (Promega, Madison, WI, USA) for 30 min, and reverse-transcribed using M-MLV Reverse Transcriptase (Promega). SYBR Green real-time polymerase chain reaction (PCR) was performed using the ChamQ Universal SYBR^®^ qPCR Master Mix (Vazyme Biotech Co., Ltd.) and ABI 7500 FAST sequence detection system (Life Technologies). The expression levels of all samples were normalized to the signal generated by glyceraldehyde-3-phosphate dehydrogenase. The primer sequences used were as follows: GAPDH: 5’-AACGGGAAGCTTGTCATCAA-3’ and 5’-TGGACTCCACGACGTACTCA-3’; LSD1: 5’-GTGGACGAGTTGCCACATTTC-3’ and 5’-TGACCACAGCCATAGGATTCC-3’; PRAC1: 5’-GCCCATTTCTCAGATCAAGG-3’ and 5’-GGTCTCGCCCAGTAGATGTT-3’.

## Western blotting analysis

Cells were lysed using radioimmunoprecipitation assay buffer supplemented with protease inhibitor cocktail tablets (Roche, Basel, Switzerland), and the total protein content was measured using a BCA kit (Beyotime Biotechnology, Shanghai, China). Thirty to fifty micrograms of total protein were resolved using sodium dodecyl sulfate polyacrylamide gel electrophoresis and transferred to a PVDF membrane (Bio-Rad Laboratories, Hercules, CA, USA). The membrane was sequentially incubated with primary antibodies and horseradish peroxidase (HRP)-conjugated secondary antibody, followed by incubation with Clarity Western enhanced chemiluminescence substrate (Bio-Rad Laboratories). Protein signals were then visualized by exposing the membrane to X-ray films (FujiFilm, Tokyo, Japan) in a dark room. The primary antibodies used were anti-GAPDH (Abcam, Waltham, MA, USA) and anti-LSD1 (Abcam).

### Cell proliferation assay

Cells were seeded into 96-well plates (2 × 103 cells/well), and cell proliferation was assessed using CCK-8 (MedChemExpress) and the BeyoClick™ EdU Cell Proliferation Kit (Beyotime Biotechnology) according to the manufacturers’ instruction.

### Luciferase assays

Cells were co-transfected with 0.5 µg of firefly luciferase reporter vector, 0.05 µg of Renilla luciferase control vector (pRL-CMV), and 0.5 µg of LSD1 expression plasmid or empty vector using Lipofectamine 8000 in a 24-well plate. Luciferase assays were performed 48 h after transfection using the dual-luciferase reporter assay system (Promega). Firefly luciferase activity was normalized to the Renilla luciferase activity.

### Cell migration and invasion assays

Cell migration and invasion were assessed using Transwell assays. Inserts with 8-µm pores, either uncoated (for migration) or coated with Matrigel (for invasion; Costar, High Wycombe, UK), were placed into wells of a 24-well plate. The lower chamber was filled with 600 µL of RPMI 1640 medium supplemented with 10% fetal bovine serum. LNCaP cells were washed once with Hank’s Balanced Salt Solution and resuspended in 100 µL of serum-free RPMI 1640 medium at a density of 1 × 10⁵ cells, which were then seeded into the upper chamber. Following an 18-h incubation at 37 °C at 5% CO₂, non-migrated cells on the upper surface of the membrane were carefully removed using a cotton swab. Cells that had migrated to the lower side of the membrane were fixed with cold methanol for 10 min and subsequently stained with 0.01% crystal violet in 20% ethanol.

### Scratch wound healing assay

LNCaP cells were seeded in a culture plate and allowed to form a confluent monolayer. A straight scratch was then carefully created in the cell layer using a sterile pipette tip. The detached cells were washed away with phosphate-buffered saline (PBS), and fresh medium was added. The plate was placed in an incubator, and images of the scratch were captured at 0 and 24 h using a microscope.

### Enzyme-Linked Immunosorbent Assays

Given its size of 6 kDa, the PRAC1 protein is difficult to detect using western blotting. Therefore, we performed enzyme-linked immunosorbent assays (ELISA) to measure PRAC1 protein expression. To this end, we coated the wells of a microtiter plate with anti-PRAC1 antibody (abx310485, Abbexa Ltd, Cambridge, UK) at a concentration of 10 µg/ml. After blocking, we added 100 µL of total cell lysate (1 µg/µL) to each well and incubated the plate at room temperature for 2 h. The plate was washed three times with PBS, 100 µL of HRP-conjugated anti-PRAC1 antibody was added (abx310486, Abbexa Ltd), and the plate was incubated at room temperature for 2 h. The plate was washed three times with PBS, and substrate working solution was prepared using the SignalUp™ Super Sensitive ELISA Assay Kit with Fluorescent HRP Substrate according to the manufacturer’s instructions (Beyotime Biotechnology). One hundred microliters of this substrate working solution was then added to each well, and the fluorescence intensity was measured using a microplate reader.

### Gene expression analysis

The expression levels of PRAC1 and LSD1 and their correlation in cancerous and non-cancerous prostate tissues were retrieved from the Gene Expression Profiling Interactive Analysis (http://gepia.cancer-pku.cn/index.html) database^34^. Genes potentially regulating PRAC1 expression were identified by searching the GPSAdb database (https://www.gpsadb.com/).

### Statistical analysis

Data are presented as the mean ± SD of values from at least three independent experiments. Statistical analyses were performed using Student’s t-test. A p-value < 0.05 was considered to indicate statistically significant intergroup differences. Statistical analyses were conducted using GraphPad Prism 8 (GraphPad Software Inc., San Diego, CA, USA; RRID: SCR_002798).

## Supplementary Information

Below is the link to the electronic supplementary material.

Supplementary Material 1

Supplementary Material 2

## Data Availability

The datasets generated during and/or analyzed in the current study are available from the corresponding author on reasonable request.
