# Supplementary material for: LSD1 promotes prostate cancer cell proliferation by upregulating PRAC1 expression
Source: Sci Rep. 2026 Mar 10;16:12974. doi: 10.1038/s41598-026-42928-8 (PMC13096199; doi:10.1038/s41598-026-42928-8)

**LSD1 promotes prostate cancer cell proliferation by upregulating *PRAC1* expression**

Yang Liao[1], Chuan Liu[1*]

Full length western blot image for Fig. 4B

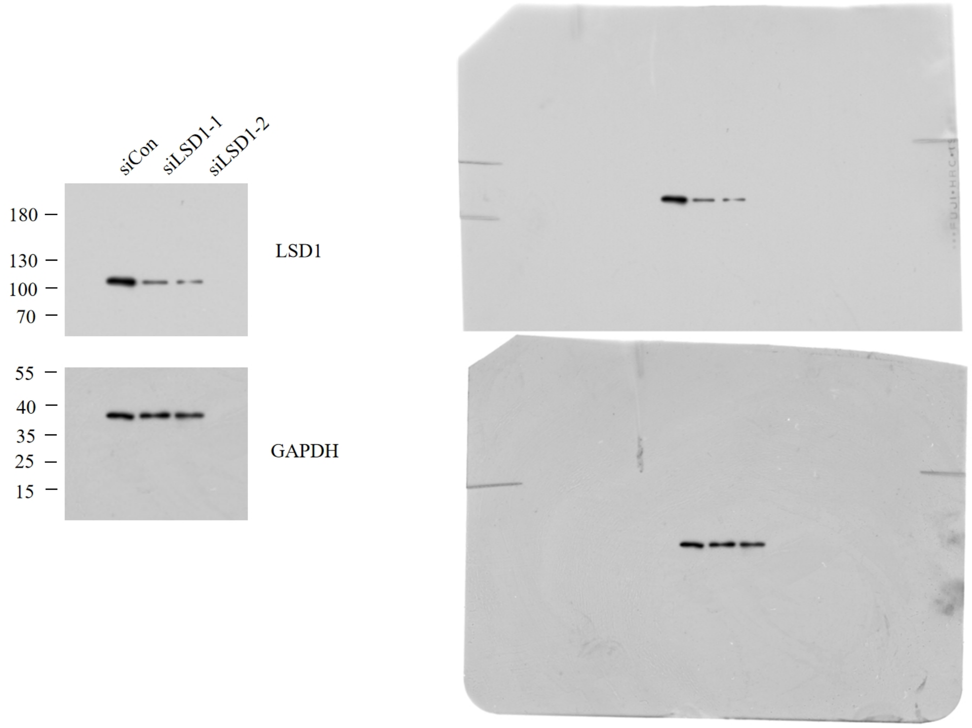


Full length western blot image for Fig. 5C

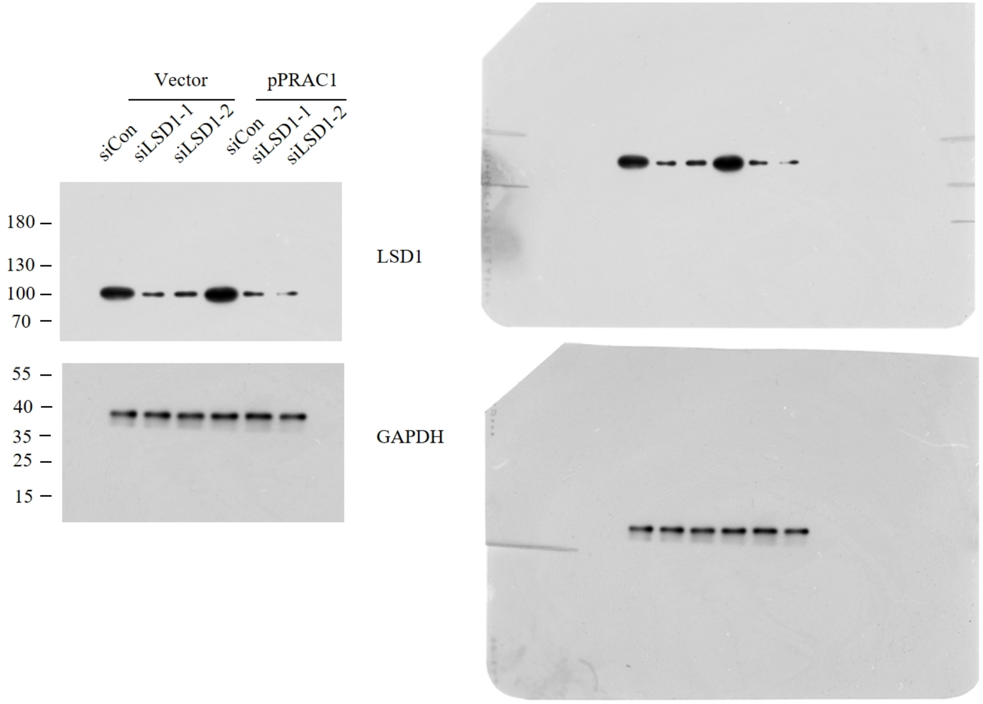

Full length western blot image for Fig. S2B

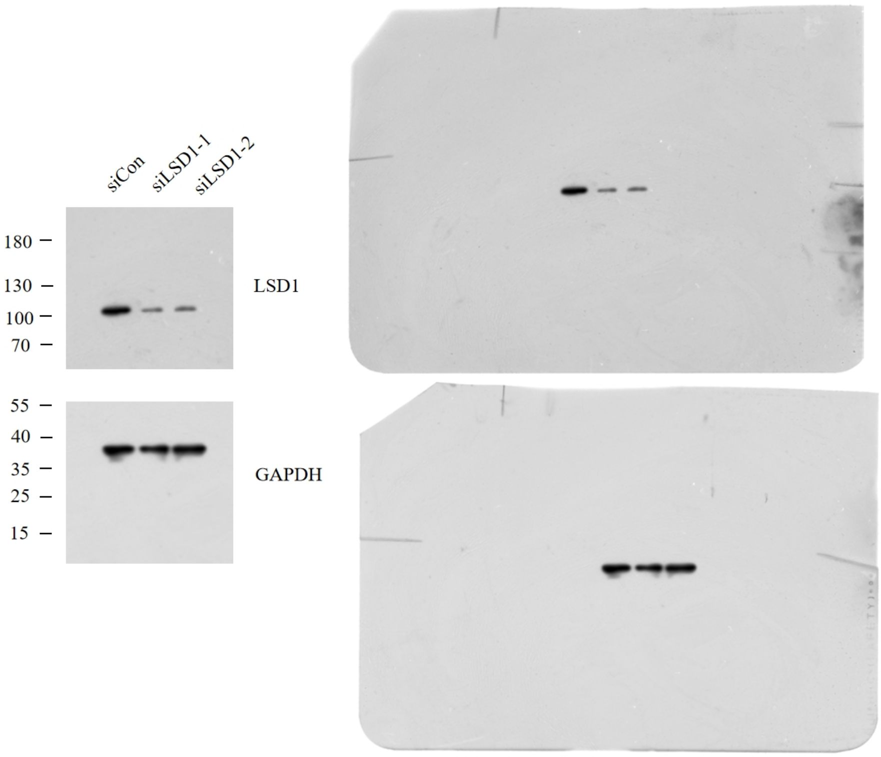

Supplement: Supplementary file 1 — Supplementary Material 1 [file 41598_2026_42928_MOESM1_ESM.pdf]
