# Supplementary material for: LSD1 promotes prostate cancer cell proliferation by upregulating PRAC1 expression
Source: Sci Rep. 2026 Mar 10;16:12974. doi: 10.1038/s41598-026-42928-8 (PMC13096199; doi:10.1038/s41598-026-42928-8)
Supplement: Supplementary file 2 — Supplementary Material 2 [file 41598_2026_42928_MOESM2_ESM.pdf]

LSD1 promotes prostate cancer cell proliferation by upregulating *PRAC1*

Yang Liao[1], Chuan Liu[1*]

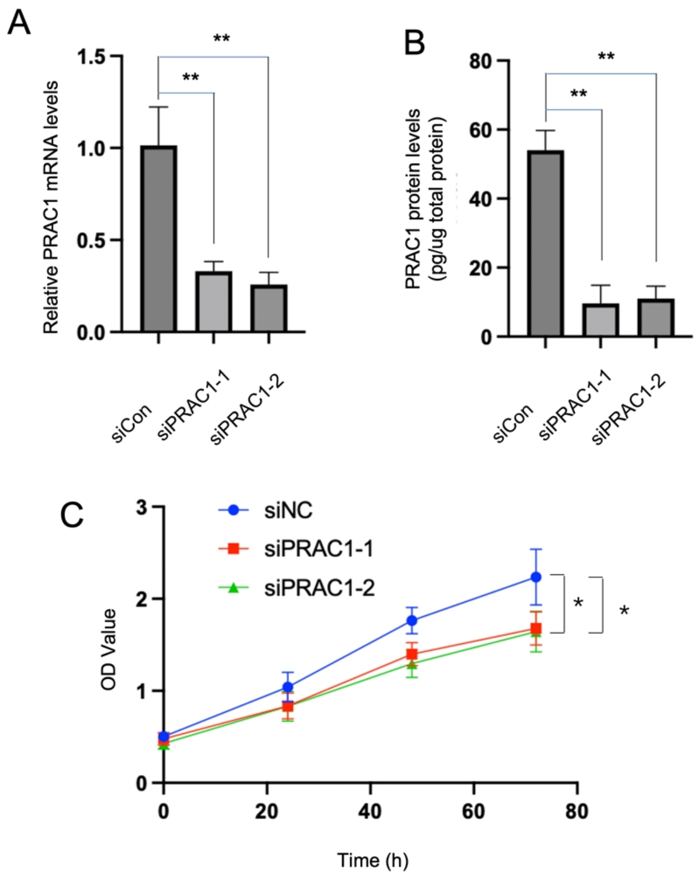

Fig. S1 PRAC1 knockdown inhibits prostate cancer cell proliferation.

DU145 cells were transfected with control or PRAC1 siRNAs, the mRNA (A) and protein (B) levels of PRAC1 were determined using reverse transcription quantitative PCR (RT-qPCR) and enzyme-linked immunosorbent assays (ELISA), respectively, at 48 h after transfection. (C) Cell proliferation assays were performed at different time points after transfection. Statistical analysis was performed using two-sided Student's *t*-tests; $*p < 0.05$, $** p < 0.001$.

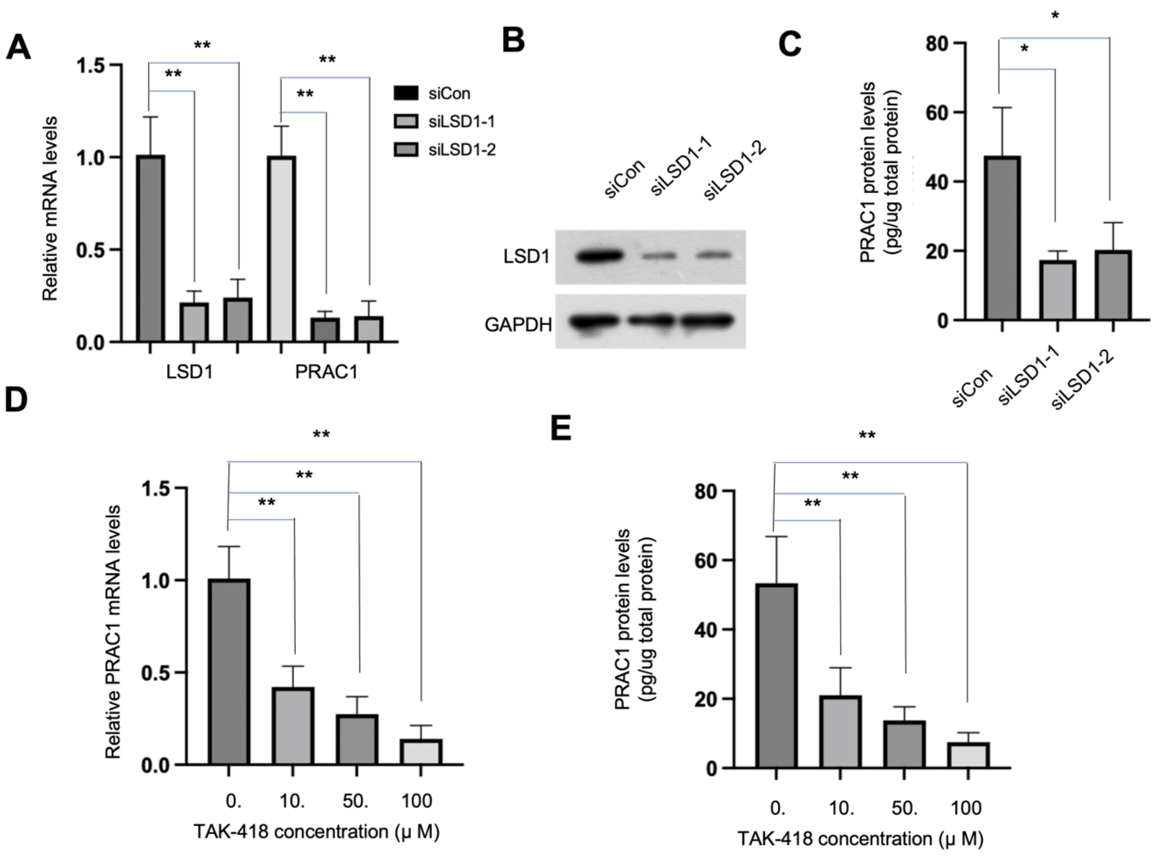

Fig. S2 LSD1 promotes PRAC1 expression in prostate cancer cells.

(A-C) DU145 cells were transfected with control or LSD1 siRNAs, (A) LSD1 and PRAC1 mRNA levels were determined using RT-qPCR 48 h after transfection, (B) LSD1 protein levels were determined using western blotting 48 h after transfection, (C) PRAC1 protein levels were determined using ELISA 48 h after transfection. (D,E) PRAC1 mRNA (D) and protein (E) levels in DU145 cells were determined using reverse transcription quantitative PCR and ELISA, respectively, at 72 h after treatment with different doses of TAK418.
